# Numerical modeling of a multi-frequency receiving system based on an array of dipole antennas for LSPE-SWIPE

**DOI:** 10.3762/bjnano.13.77

**Published:** 2022-09-01

**Authors:** Alexander V Chiginev, Anton V Blagodatkin, Dmitrii A Pimanov, Ekaterina A Matrozova, Anna V Gordeeva, Andrey L Pankratov, Leonid S Kuzmin

**Affiliations:** 1 Nizhny Novgorod State Technical University, Nizhny Novgorod, Minin Street, 24, 603950, Russia; 2 Institute for Physics of Microstructures of the Russian Academy of Sciences, GSP-105, Nizhny Novgorod, 603950, Russia; 3 Chalmers University of Technology, Department of Microtechnology and Nanoscience - MC2, Gothenburg, SE-412 96, Sweden

**Keywords:** cosmic microwave background (CMB), cold-electron bolometer, dichroic antenna, dipole antenna, LSPE-SWIPE, waveguide horn

## Abstract

Here we present the results of a numerical modeling of mode composition in the constriction of the Large Scale Polarization Explorer-Short-Wavelength Instrument for the Polarization Explorer (LSPE-SWIPE) back-to-back horn. These results are used for calculating the frequency response of arrays of planar dipole antennas with cold-electron bolometers for 145, 210, and 240 GHz frequencies. For the main frequency channel (i.e., 145 GHz) we have a 45 GHz bandwidth. For the auxiliary frequency channels (i.e., 210 and 240 GHz) placed on the same substrate, we have bandwidths of 26 and 38 GHz, respectively. We performed some optimizations for cold-electron bolometers to achieve a photon noise-equivalent power of 1.1 × 10^−16^ W/Hz^1/2^. This was achieved by replacing one of two superconductor–insulator–normal tunnel junctions with a superconductor–normal metal contact.

## Introduction

The Large Scale Polarization Explorer (LSPE) [1] is an experiment of the Italian Space Agency for observing the polarization pattern of the B-mode of the cosmic microwave background (CMB). These observations can give an important information about primordial gravitational waves. The LSPE project consists of two instruments: Strip and Short-Wavelength Instrument for the Polarization Explorer (SWIPE). LSPE-SWIPE is a balloon-borne radio telescope, which will consist of a main and two auxiliary frequency channels. According to the latest requirements [2], the operating frequency of the LSPE-SWIPE main frequency channel should be 145 GHz with a bandwidth of 30%. The operating frequencies of the auxiliary channels should be equal to 210 and 240 GHz and the bandwidths should be 20% and 10%, respectively. To receive radiation in the indicated frequency ranges, we propose to use arrays of planar dipole antennas located in the opening of a special bidirectional horn that forms the radiation pattern of the receiving system and also serves as a low-frequency filter.

For radiation detection, many bolometer types can be applied. The detectors for this instrument should work at 300 mK, since this is the working temperature of the ^3^He cryostat used for the LSPE project. One of main candidates for LSPE-SWIPE is a transition-edge sensor (TES) with a spiderweb antenna [2–3]. For the OLIMPO mission, kinetic inductance detectors (KIDs) were used [4]. We propose to use cold-electron bolometers (CEBs) [5–6] integrated into the dipole antennas. The advantages of CEB over other types of bolometers are, in particular, their high sensitivity with background-limited operation [6–8] and a wide dynamic range. These qualities are largely determined by the presence of an internal self-cooling of the electronic subsystem of the CEB absorber [6–8]. Another key advantage for balloon and space missions is the high immunity of CEB against cosmic rays [9] due to a double protection given by the extremely small volume of the absorber and decoupling of electron and phonon systems.

One of the advantages of our implementation is the possibility to make arrays for two auxiliary 210 and 240 GHz channels on the same pixel due to the small size of CEBs. The use of multichroic pixels increases the number of pixels per frequency, at no extra cost per focal plane area, weight, and cryogenic load. Before, the double-frequency system with slot antennas and CEBs for 75 and 105 GHz frequencies for the Cosmic Origins Explorer (COrE) mission has been investigated and a band separation was demonstrated [10]. Now this approach is getting further improvements with arrays of dipole antennas and a voltage-biased mode. To use this mode, the CEBs must be connected in parallel. Superconducting quantum interference devices (SQUIDs) with multiplexing [11] will also be used for readout. Therefore, to match this readout system, the arrays of dipole antennas and CEBs connected in parallel should have a resistance value of 1 Ohm at the working point.

In the present paper, we describe the problems of numerical modeling for double-frequency arrays of dipole antennas connected in parallel on a single substrate with radiation going through the back-to-back horn with predefined parameters.

## Results

### Calculation of the mode composition in the horn constriction at the incidence plane of a linearly polarized plane wave

**Figure 1:**
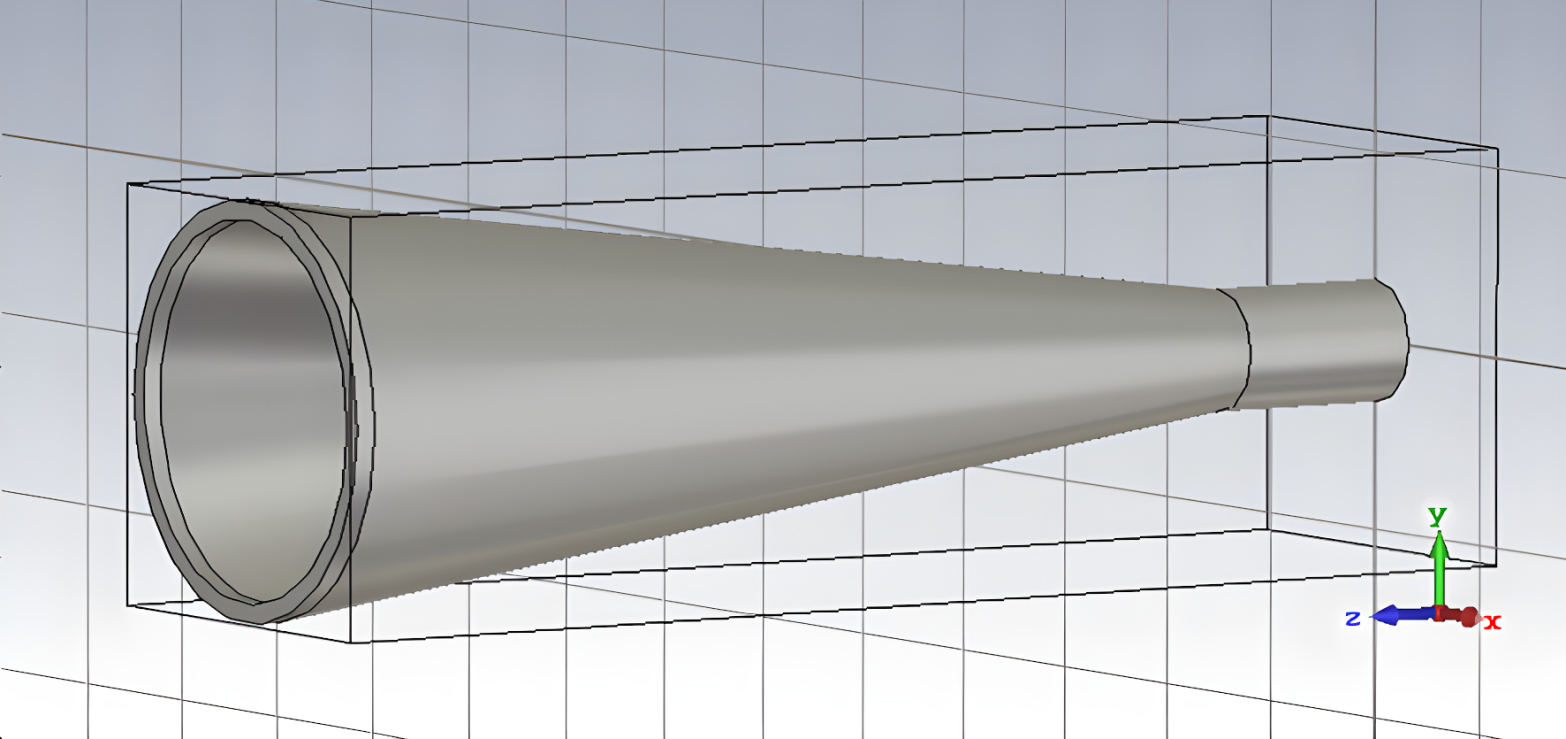
The front part and the constriction of the back-to-back horn of the LSPE-SWIPE receiving system [3] used for the calculation of the mode composition in the constriction.

The parameters of the back-to-back horn are defined in [3]. They consist of two openings and a constriction connecting them, see the details in [3] and a part of the horn in Figure 1. One of the openings defines the radiation pattern of the receiving system, and the other is facing the receiving antenna array. The constriction of the horn determines the modal composition of the electromagnetic field passing through the horn. For a given constriction diameter, up to 38 modes of a circular waveguide are propagating in the operating frequency range.

For correct numerical modeling of the frequency response of the receiving system, it is necessary to know the distribution of the field of the incident wave on the matrix of receiving cells. The most consistent would be modeling a back-to-back horn irradiated by a plane electromagnetic wave with a receiving matrix installed in the opposite opening. However, this task requires a lot of computational resources. Therefore, the problem of irradiating the receiving matrix with an incoming radiation was divided into two parts. The first problem was to determine the mode composition of the radiation passing through the constriction of a back-to-back horn when a plane electromagnetic wave falls on it (Figure 1). The second problem was to calculate the power absorbed by the receiving matrix when this matrix is irradiated by the electromagnetic field of a round waveguide port with a calculated mode composition.

**Figure 2:**
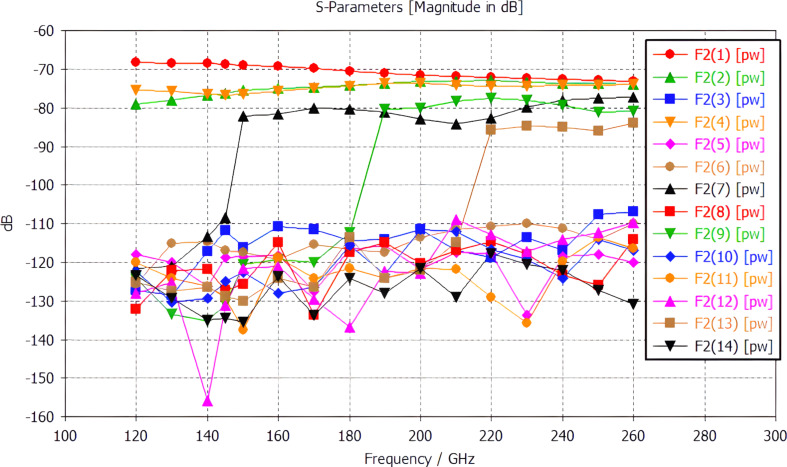
The mode composition of the electromagnetic field in the constriction of a bidirectional horn as a function of frequency.

The result of the calculation of the mode composition in a back-to-back horn is shown in Figure 2. It can be seen that over the entire operating frequency range of the LSPE-SWIPE, modes with numbers 1 (main), 2, and 4 are present in the horn. Starting from the frequency of 150 GHz, mode 7 appears; amplitudes of modes 7, 9, and 13 are associated with the achievement of the corresponding cutoff frequencies of these modes. The amplitudes of the remaining modes are negligible compared to the amplitudes of the listed modes and can be ignored. The calculated mode amplitudes can be used to calculate the frequency response of antenna arrays with integrated bolometers for the main and auxiliary frequency channels of the LSPE-SWIPE.

### Calculation of the frequency response of a planar antenna matrix with integrated cold-electron bolometers

The frequency response of the receiving matrix was calculated as follows. The radiation incident on the matrix is formed by the waveguide port of a circular waveguide with a diameter corresponding to the wide part of the bidirectional horn facing the receiving system (on the right in Figure 1). An RC circuit was used as an equivalent circuit for CEB at high frequencies. In the process of numerical simulation, we calculated the dependence of the power, *P*_i_, released on the active resistance of the *i*-th RC-chain of the array of receiving cells, on the frequency of the incident radiation, and the total power in all receiving cells:


[1] $$P\left(f\right)=\sum_{i=1}^{N}P_{i}\left(f\right).$$

The summation was performed over the cells included in one frequency channel. Thus, as a result of the calculation, the frequency response of the receiving system of one frequency channel was obtained. This calculation principle was used for all types of receiving matrices of the LSPE-SWIPE auxiliary frequency channels.

### Main frequency channel of the LSPE-SWIPE

**Figure 3:**
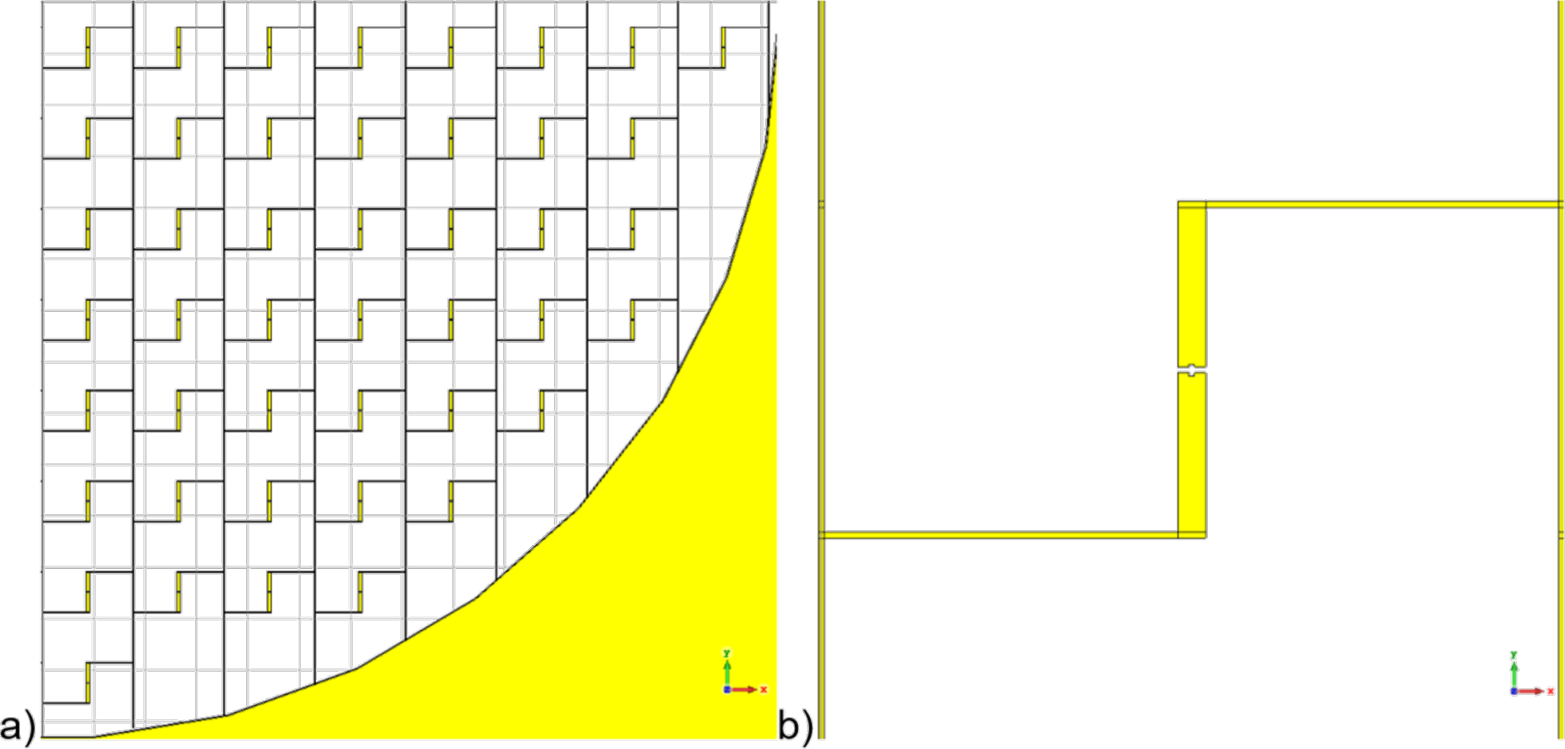
Receiving system of the LSPE-SWIPE145 GHz main channel. a) A quarter of receiving cells matrix on the 14 mm chip. b) A single cell of the receiving system based on a dipole antenna.

The LSPE-SWIPE 145 GHz main channel receiving system is based on an array of dipole antennas with integrated CEBs (Figure 3). The receiving cells are located within a circle under the opening of a bidirectional horn. Its characteristic feature is that the antenna dipoles and wires that provide the CEB DC bias are located in the same layer. This significantly simplifies and reduces the cost of manufacturing such structures.

**Figure 4:**
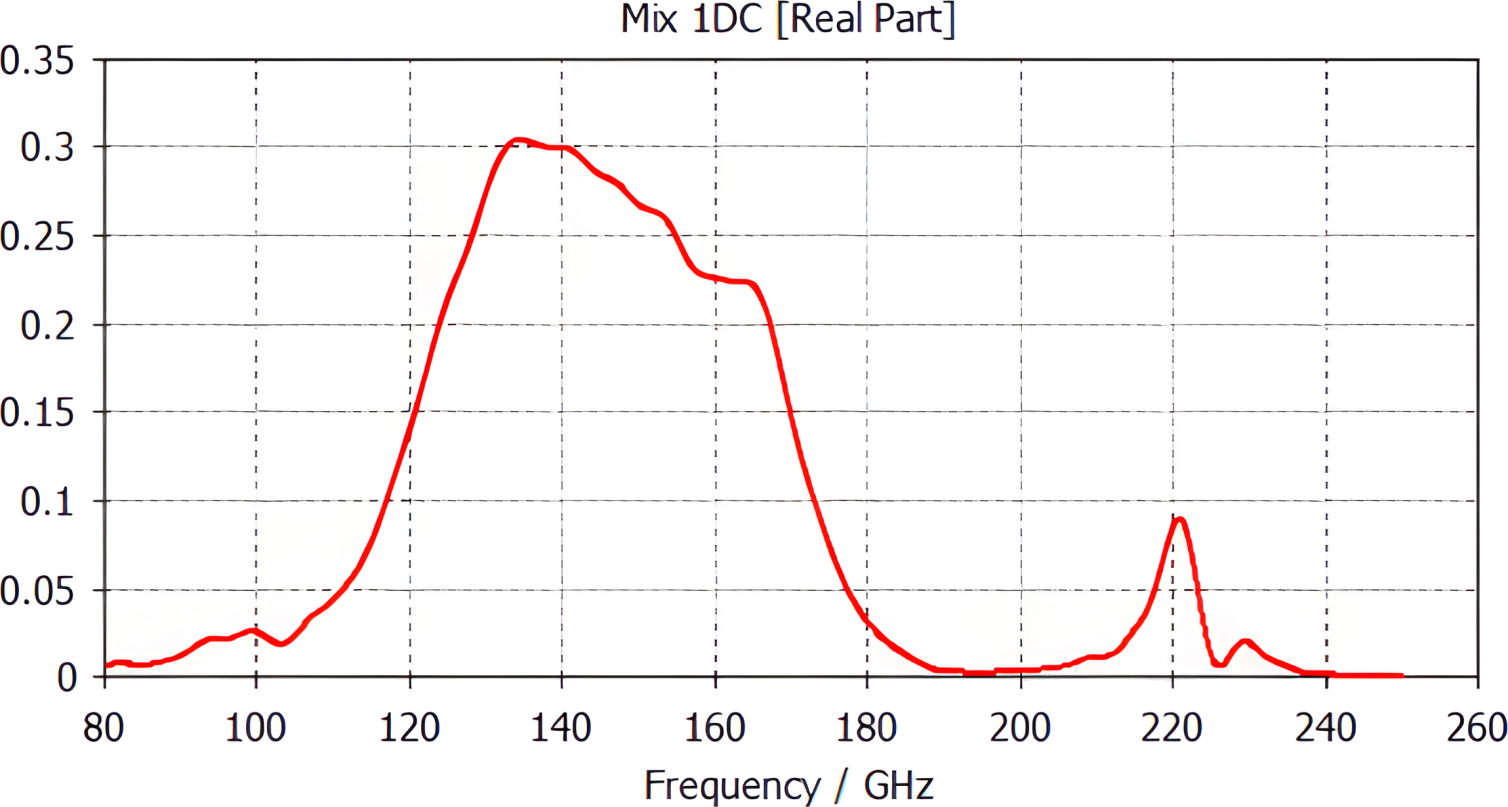
Frequency response of the LSPE-SWIPE 145 GHz main frequency channel.

The frequency response of the LSPE-SWIPE 145 GHz main frequency channel is shown in Figure 4. The received frequency band of the main frequency channel at the level of 0.5 is 45 GHz. At 220 GHz there is a spurious resonance that can be suppressed with a low-pass filter.

### Auxiliary frequency channels of the LSPE-SWIPE

The cells are located within a circle with a diameter of 4.5 mm; while one half of the circle is occupied by cells for 210 GHz, the second half is occupied by cells for 240 GHz. Receiving cells with integrated CEBs are located on a 260 µm thick silicon substrate with a silicon dioxide layer. The difficulty of the considered receiving system is the existing specific sample holder, which allows the receiving of the signal from the front side of the pixel only with the back-short placed at the back side of the pixel. Such configuration leads to tougher constraints and less number of free parameters than in [10], where the signal was coming to the antennas through the substrate.

**Figure 5:**
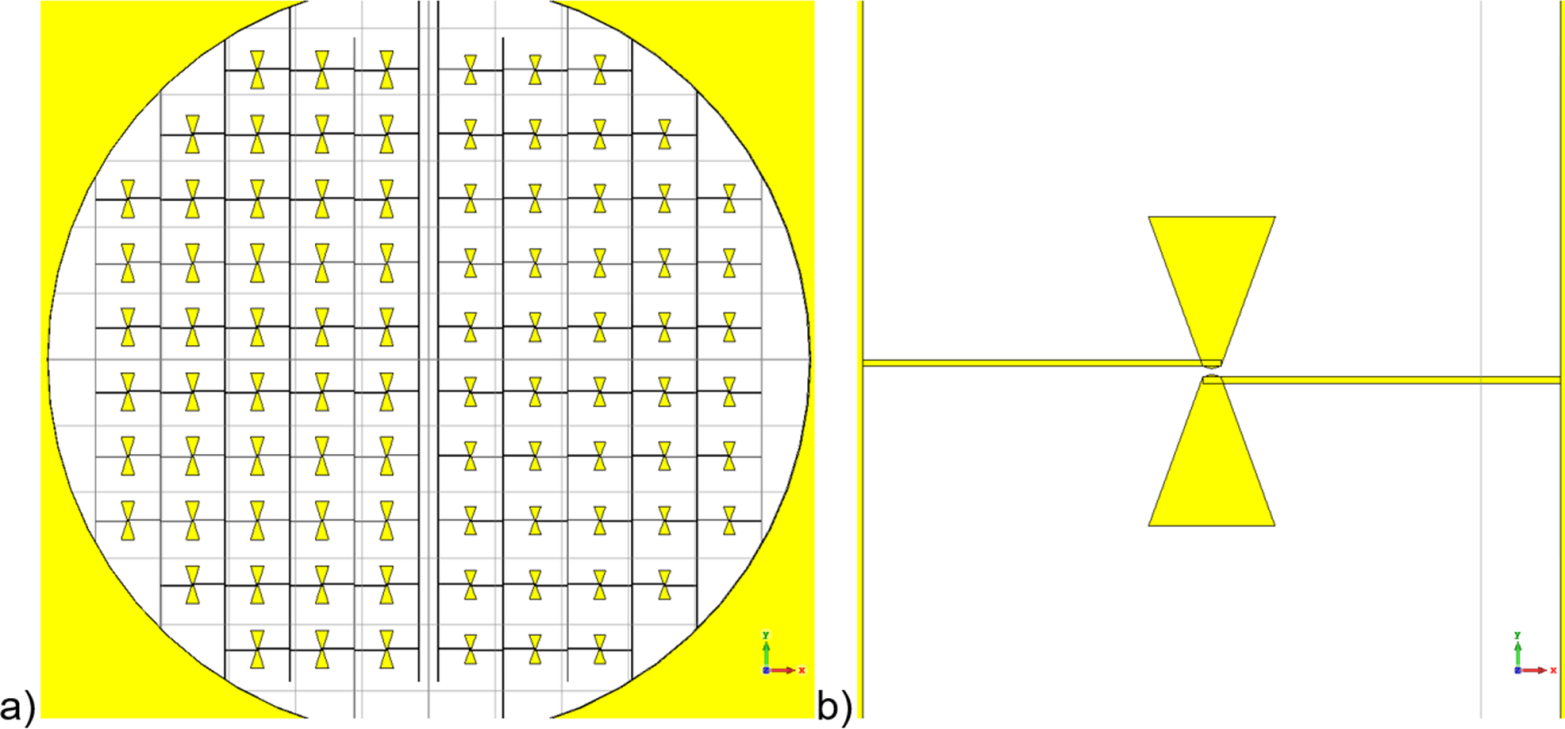
a) Receiving cell array based on bow-tie antennas; half of the 7 mm plate on the left are 210 GHz and on the right 240 GHz frequency channels. b) A receiving system cell based on a bow-tie antenna.

In this work, the receiving system based on a bow-tie dipole antenna was calculated (Figure 5) with the DC bias lines connected to the central parts of the antennas.

**Figure 6:**
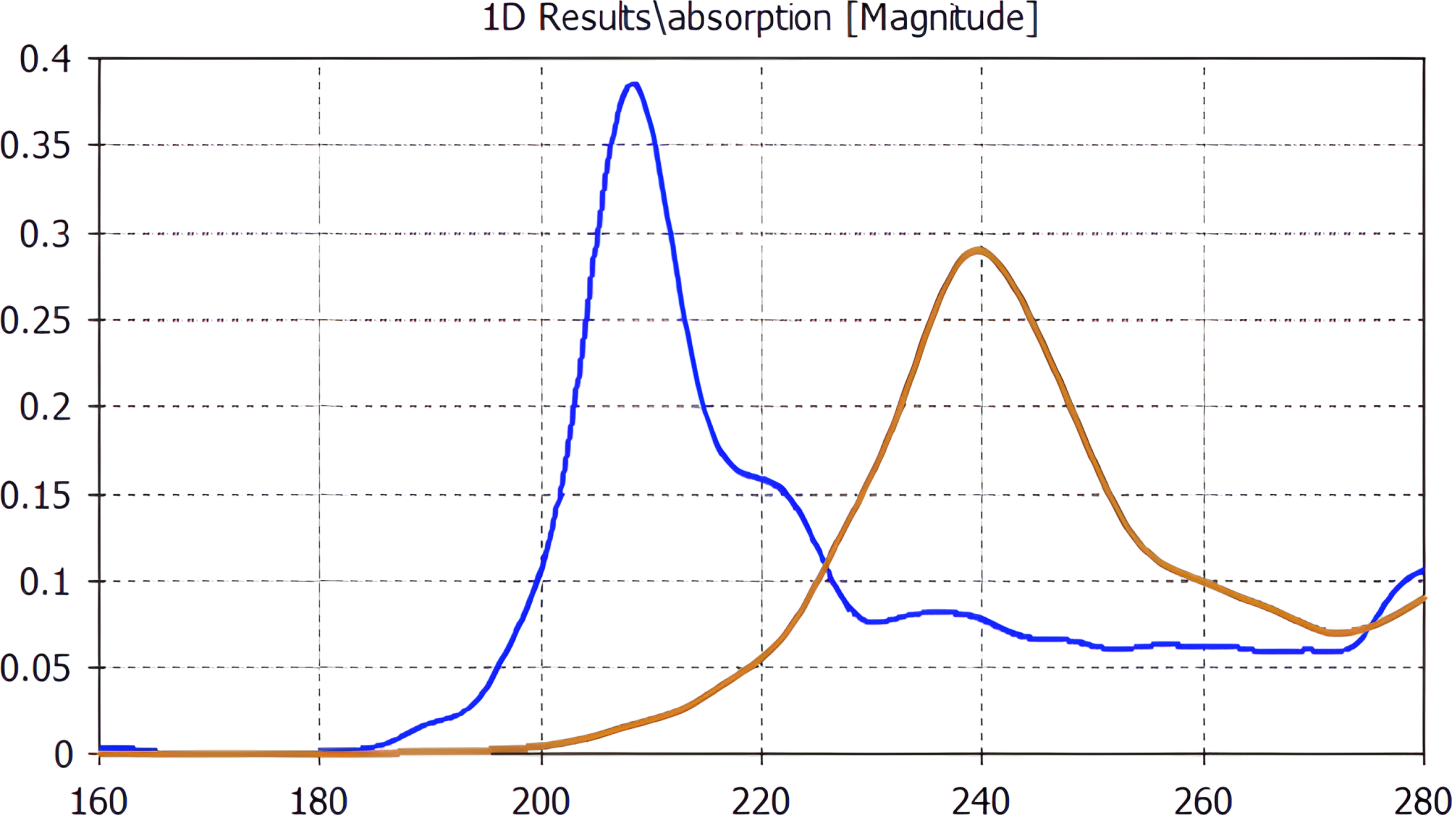
Frequency response of a matrix of receiving cells based on bow-tie antennas for 210 GHz and 240 GHz frequency channels.

The frequency response of the receiving matrix with bow-tie antennas is shown in Figure 6. Rather good band separation is visible in spite of a certain cross-talk of the 210 GHz channel. The frequency response width at 50% of the maximum level for the 210 GHz channel is 26 GHz and for the 240 GHz channel is 38 GHz. In addition, the radiofrequency (RF) tails of the 240 GHz channel above 270 GHz are supposed to be suppressed by a band-pass filter.

### Noise-equivalent power calculations for the LSPE-SWIPE

The noise-equivalent power (NEP) is a measure of a minimal signal that can be detected, and it qualifies the sensitivity of a detector. The NEP is the ratio of the total system noise (which includes both the internal noise of the detector and the photon noise of the received signal, depending on the signal power) with respect to the responsivity, which can be calculated using the heat balance equations [6].

The power load for the LSPE-SWIPE 145, 210, and 240 GHz frequency channels should be 11, 12.4, and 16 pW, respectively, as stated in Table 4 in [2]. The total NEP level should be approx. 7 × 10^−17^ W/Hz^1/2^ for the 145 GHz channel, 8.5 × 10^−17^ W/Hz^1/2^ for the 210 GHz channel, and 1.3 × 10^−16^ W/Hz^1/2^ for the 240 GHz channel at the working point for a background (photon-noise) limited operation. However, the readout system is based on SQUIDs, and this system has an input current noise of 4–10 pA/Hz^1/2^. To optimize the receiver for better noise characteristics, we consider optimized CEBs with a single superconductor–insulator–normal (SIN) tunnel junction and a single superconductor–normal (SN) contact [12]. Combined together, they form a SINS structure.

This solution can help reaching better noise characteristics than those of CEBs with two SIN tunnel junctions due to several reasons. First, the responsivity is increased by a factor of two due to hot electrons tunneling only through one SIN junction. Second, the bolometer resistance is decreased twice, which helps in array matching with a SQUID readout (the total resistance of this array should be 1 Ohm). Third, the electron cooling efficiency is increased by a factor of two due to the two-fold increase in the readout for the same power going through the system. Last but not least, the absence of a second SIN tunnel junction suppresses the Coulomb blockade, so the absorber volume can be decreased by a factor of four, leaving the capacitance unchanged.

Therefore, the current responsivity is increased from 40–45 nA/pW to 80–100 nA/pW. Thus, the total NEP for this CEB concept should also be two times better than the total NEP for CEBs with two SIN tunnel junctions and it should be close to the required photon NEP level for the LSPE-SWIPE frequency channels. This comparison for arrays of 66 SINS CEBs with a 6 pW power load is shown in Figure 2 in [12].

For better NEP estimation we should also account for the matching of the CEB array with the SQUID readout system. The mismatching can happen if there is dynamic resistance of the CEB array at the working point with a parasitic resistance of connecting wires higher than 1 Ohm. Then the noise of the SQUID readout system is multiplied by the square root of the ratio of the obtained dynamic resistance of the CEB array and the connecting wires to the required value of 1 Ohm. The NEP estimations for different numbers of CEBs in a parallel array for 145, 210, and 240 GHz frequency channels accounting for possible mismatching with the SQUID readout system are shown below with a SQUID noise of 10 pA/Hz^1/2^.

**Figure 7:**
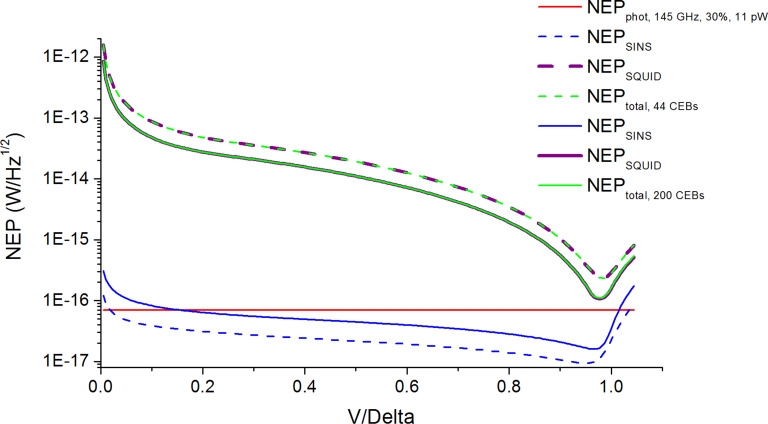
NEP estimations for the 145 GHz channel with 11 pW power load. Dashed curves are for 44 SINS CEBs; solid curves are for 200 SINS CEBs.

**Figure 8:**
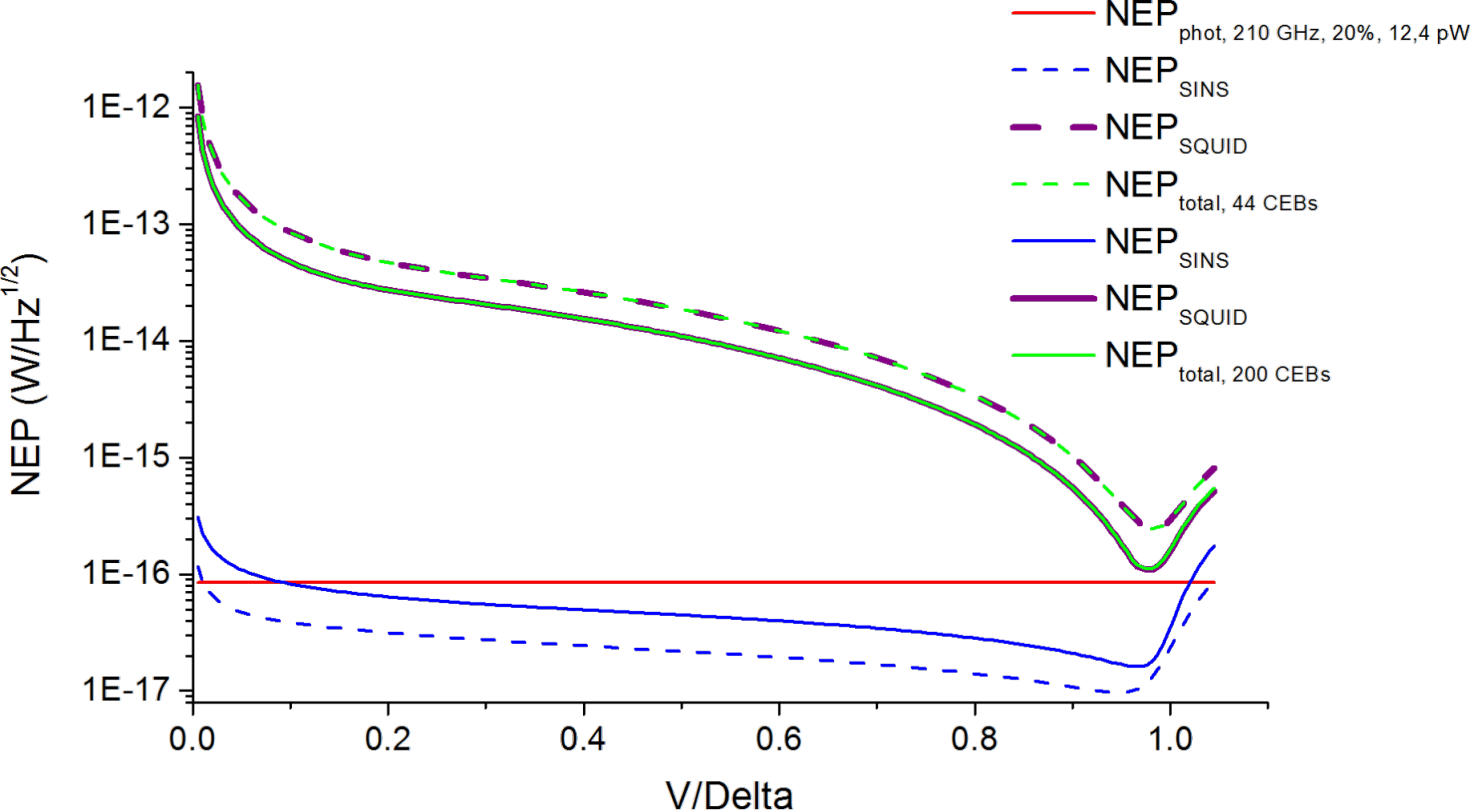
NEP estimations for the 210 GHz channel with 12.4 pW power load. Dashed curves are for 44 SINS CEBs; solid curves are for 200 SINS CEBs.

**Figure 9:**
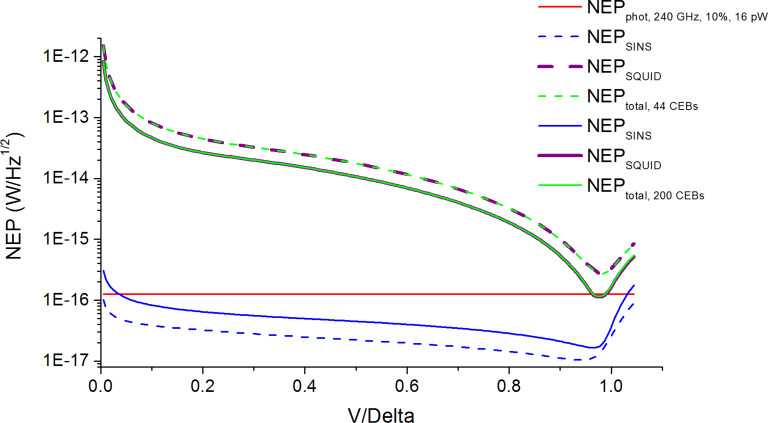
NEP estimations for the 240 GHz channel with 16 pW power load. Dashed curves are for 44 SINS CEBs; solid curves are for 200 SINS CEBs.

In Figure 7 and Figure 8 it is seen that the total NEP level (green curves), which includes the NEP of SINS junctions (blue curves) and the NEP of the SQUID readout system (purple curves), nearly approaches the photon NEP level (red line), so the increase in the number of CEBs improves the total NEP. For 200 SINS CEBs of the 240 GHz frequency channel (Figure 9, solid curves), the total NEP curve reaches the photon NEP line at the working point. The minimal value of total NEP in this case is 1.15 × 10^−16^ W/Hz^1/2^.

These estimations were calculated for an input current noise of the SQUID readout system in the worst case scenario of 10 pA/Hz^1/2^. If this noise is reduced twice (i.e., to 5 pA/Hz^1/2^) the minimal value of the total NEP for 200 SINS CEBs of 240 GHz frequency channel falls below 7 × 10^−17^ W/Hz^1/2^. In addition, the photon-noise limit can be easily reached for all frequency channels with the use of the Quasiparticle Cascade Amplifier (QCA), which can be added to the SINS CEB. The great benefits of using the QCA are described in detail in [12].

## Conclusion

The calculation of the mode composition in the constriction of the LSPE-SWIPE back-to-back horn helps us in modeling the frequency response of the receiving systems. We use the calculated mode amplitudes for investigating the frequency response of the arrays of dipole antennas for all frequency channels of the LSPE-SWIPE receiving system.

We have developed a theoretical model of arrays of dipole antennas which can be applicable for all frequency channels of the LSPE-SWIPE. For this purpose, we have used both straight dipole antennas and the bow-tie shaped dipole antennas, which are very useful for meeting the bandwidth requirements for different frequency channels.

To achieve NEP level for photon-noise limited operations, we have performed a very important optimization for cold-electron bolometer design. We changed one of two SIN tunnel junctions to the SN contact, so it became a SINS structure. This change allowed us to increase current responsivity twice and to improve total NEP by the same factor. However, further improvements can be achieved by the use of the Quasiparticle Cascade Amplifier [12].

## References

[1] Aiola S, Amico G, Battaglia P, Battistelli E, Baù A, de Bernardis P, Bersanelli M, Boscaleri A, Cavaliere F, Coppolecchia A (2012). Proc SPIE.

[2] Kase R, Tsujikawa S (2021). J Cosmol Astropart Phys.

[3] Lamagna L, Addamo G, Ade P A R, Baccigalupi C, Baldini A M, Battaglia P M, Battistelli E, Baù A, Bersanelli M, Biasotti M (2020). J Low Temp Phys.

[4] Masi S, de Bernardis P, Paiella A, Piacentini F, Lamagna L, Coppolecchia A, Ade P A R, Battistelli E S, Castellano M G, Colantoni I (2019). J Cosmol Astropart Phys.

[5] Kuzmin L, Pekola J, Ruggiero B, Silvestrini P (2002). Optimization of the Hot-Electron Bolometer and A Cascade Quasiparticle Amplifier for Space Astronomy. International Workshop on Superconducting Nano-Electronics Devices.

[6] Kuzmin L S, Pankratov A L, Gordeeva A V, Zbrozhek V O, Shamporov V A, Revin L S, Blagodatkin A V, Masi S, de Bernardis P (2019). Commun Phys.

[7] Gordeeva A V, Zbrozhek V O, Pankratov A L, Revin L S, Shamporov V A, Gunbina A A, Kuzmin L S (2017). Appl Phys Lett.

[8] Gordeeva A V, Pankratov A L, Pugach N G, Vasenko A S, Zbrozhek V O, Blagodatkin A V, Pimanov D A, Kuzmin L S (2020). Sci Rep.

[9] Salatino M, de Bernardis P, Kuzmin L S, Mahashabde S, Masi S (2014). J Low Temp Phys.

[10] Kuzmin L S, Blagodatkin A V, Mukhin A S, Pimanov D A, Zbrozhek V O, Gordeeva A V, Pankratov A L, Chiginev A V (2019). Supercond Sci Technol.

[11] Heinz E, Zakosarenko V, May T, Meyer H G (2013). Supercond Sci Technol.

[12] Kuzmin L, Golubev D S (2022). IEEE Trans Appl Supercond.

